# Trace metal content prediction along an AMD (acid mine drainage)-contaminated stream draining a coal mine using VNIR–SWIR spectroscopy

**DOI:** 10.1007/s10661-023-11837-y

**Published:** 2023-10-02

**Authors:** Jamie-Leigh Robin Abrahams, Emmanuel John M. Carranza

**Affiliations:** Department of Geology, Faculty of Natural and Agricultural Sciences, University of the Free State, 205 Nelson Mandela Drive, Park West, Bloemfontein, 9301 South Africa

**Keywords:** Remote sensing, Reflectance spectroscopy, Floodplain sediments, Heavy metal, Predictive modeling

## Abstract

The current study investigated the use of VNIR–SWIR (visible/near infrared to short-wavelength infrared: 400–2500 nm) spectroscopy for predicting trace metals in overbank sediments collected in the study site. Here, we (i) derived spectral absorption feature parameters (SAFPs) from measured ground spectra for correlation with trace metal (Pb, Cd, As, and Cu) contents in overbank sediments, (ii) built univariate regression models to predict trace metal concentrations using the SAFPs, and (iii) evaluated the predictive capacities of the regression models. The derived SAFPs associated with goethite in overbank sediments were Depth433^b^, Asym433^b^, and Width433^b^, and those associated with kaolinite in overbank sediments were Depth1366^b^, Asym1366^b^, Width1366^b^, Depth2208^b^, Asym2208^b^, and Width2208^b^. Cadmium in the overbank sediments showed the strongest correlations with the goethite-related SAFPs, whereas Pb, As, and Cu showed strong correlations with goethite- and kaolinite-related SAFPs. The best predictive models were obtained for Cu (R^2^ = 0.73, SEE = 0.15) and Pb (R^2^ = 0.73, SEE = 0.21), while weaker models were obtained for As (R^2^ = 0.46, SEE = 0.31) and Cd (R^2^ = 0.17, SEE = 0.81). The results suggest that trace metals can be predicted indirectly using the SAFPs associated with goethite and kaolinite. This is an important benefit of VNIR–SWIR spectroscopy considering the difficulty in analyzing “trace” metal concentrations, on large scales, using conventional geochemical methods.

## Introduction

Trace metal pollution in the environment is one of the major concerns related to acid mine drainage (AMD). This is because trace metals do not chemically degrade and thus, can accumulate in hazardous concentrations in the environment. The accumulation of trace metals over time poses potential threat, not only to the environment and animal species, but also to human health via contaminated soil and water resources (N’Guessan et al., 2009; Wu et al., 2005; Xie et al., 2012). Thus, the detection and monitoring of trace metals in AMD-contaminated areas is crucial for mitigation of the hazard they pose to human, societal, and environmental health (Bradshaw, 2000).

Traditional wet chemical methods for detecting and monitoring trace metal contamination in the environment involve a number of, often tedious and time-consuming, steps such as (Pandit et al., 2010; Wu et al., 2007): (i) sample collection, preservation, and preparation for analysis, (ii) destructive and costly laboratory analysis, and (iii) the continuous reproduction of geochemical maps. Thus, in cases where rapid data collection and analyses are necessary to detect and monitor trace metal contamination associated with mining accidents (Kemper & Sommer, 2002, 2003) and natural disasters in mining areas (McCarthy & Humphries, 2012), the conventional geochemical methods become inefficient and costly. Consequently, there is a need for a more efficient and cost-effective method for timeous detection and monitoring of trace metal contamination in the environment.

Reflectance spectroscopy is a promising tool that offers a non-destructive, in-situ, easily reproducible, and potentially cost-effective method for predicting trace metal concentrations in the environment (Wu et al., 2007). It is the study of surface materials’ interaction with (i.e., scattering and absorption of) light (Mustard & Glotch, 2020). The proportion of light that is scattered and absorbed is largely controlled by the chemical composition and structure of the material interacted with, thus, generating distinct spectral absorption features (SAFs) for that material (Bishop, 2020). SAFs in the visible/near infrared (VNIR) to short-wavelength infrared (SWIR) region (400–2500 nm) of the electromagnetic (EM) spectrum are known to be diagnostic of soil properties and mineralogy (Ben-Dor et al., 1999; Lilliesand et al., 2015). SAFs in this region of the EM spectrum are mainly the result of electronic transitions and overtones and combinations of fundamental molecular vibrations of the crystal lattice (Bishop, 2020).

Trace metals at concentrations below 4000 ppm are spectrally inactive in the VNIR–SWIR region of the EM spectrum (Wu et al., 2007). However, they do exhibit distinct SAFs when bound to spectrally active soil components such as clays (Clark, 1999; Van der Meer, 1999) and iron oxides (Ben-Dor et al., 1999). Clay-related SAFs are associated with molecular vibrations between 1300 and 2500 nm (Clark, 1999) while iron oxide-related SAFs are mainly the result of crystal field effects and charge transfer between transition metals and related ligands between 400 and 1200 nm of the EM spectrum (Ben-Dor et al., 1999). As trace metal cations are adsorbed to surface hydroxyl (OH) groups on clays and metal oxides, H^+^ is released, thus, decreasing the number of OH sites and increasing the number of oxygen (O) sites on the mineral surface (Schindler & Sposito, 1991). The decrease in OH and increase in O sites on oxide and clay surfaces may cause changes in the area, depth, and asymmetry of their absorption peaks (here, referred to as SAFPs), thus allowing the indirect quantification of trace metals (Choe et al., 2008).

Numerous authors have demonstrated the potential of VNIR–SWIR spectroscopy for predicting trace/heavy metal contents in agricultural and mine soils (Gholizadeh et al., 2015; Ji et al., 2010; Omran, 2016; Pandit et al., 2010; Ren et al., 2009; Sawut et al., 2018; Song et al., 2015; Tu et al., 2021; Wang et al., 2022; Xie et al., 2012; Zhang et al., 2019; Zhang et al., 2022), lake- (Jiang et al., 2018; Malley & Williams, 1997), and stream sediments (Choe et al., 2008; Piroozfar et al., 2018). However, trace/heavy metal prediction in overbank/floodplain soils and sediments has received only limited attention (Lamine et al., 2019). In addition, no research has yet investigated the potential of field VNIR–SWIR spectroscopy for predicting trace/heavy metals in sediments along streams in coal mining districts. Coal mine wastes are different to that of metal mines chiefly because coal is a sedimentary deposit which, compared to non-sedimentary metallic deposits and the common metal sulfides therein, are largely dominated by pyrite (Eby, 2004; INAP, 2009). As a result, coal mine drainage is typified by elevated iron (Fe), aluminum (Al), and manganese (Mn) and trace metal concentrations related to their sedimentary strata (INAP, 2009).

Here, we assess the feasibility of using ground (field) VNIR–SWIR spectroscopy to predict trace metal contents in overbank sediments along an AMD-contaminated stream draining a coal mine. The objectives of this study were to (i) derive SAFPs from ground spectra and relate these to trace metal contents in overbank sediments in the study site, (ii) construct univariate regression models to predict trace metal concentrations using the derived SAFPs, and (iii) evaluate the stability and predictive capacities of the regression models.

## Materials and methods

### Description of the study site

**Fig. 1:**
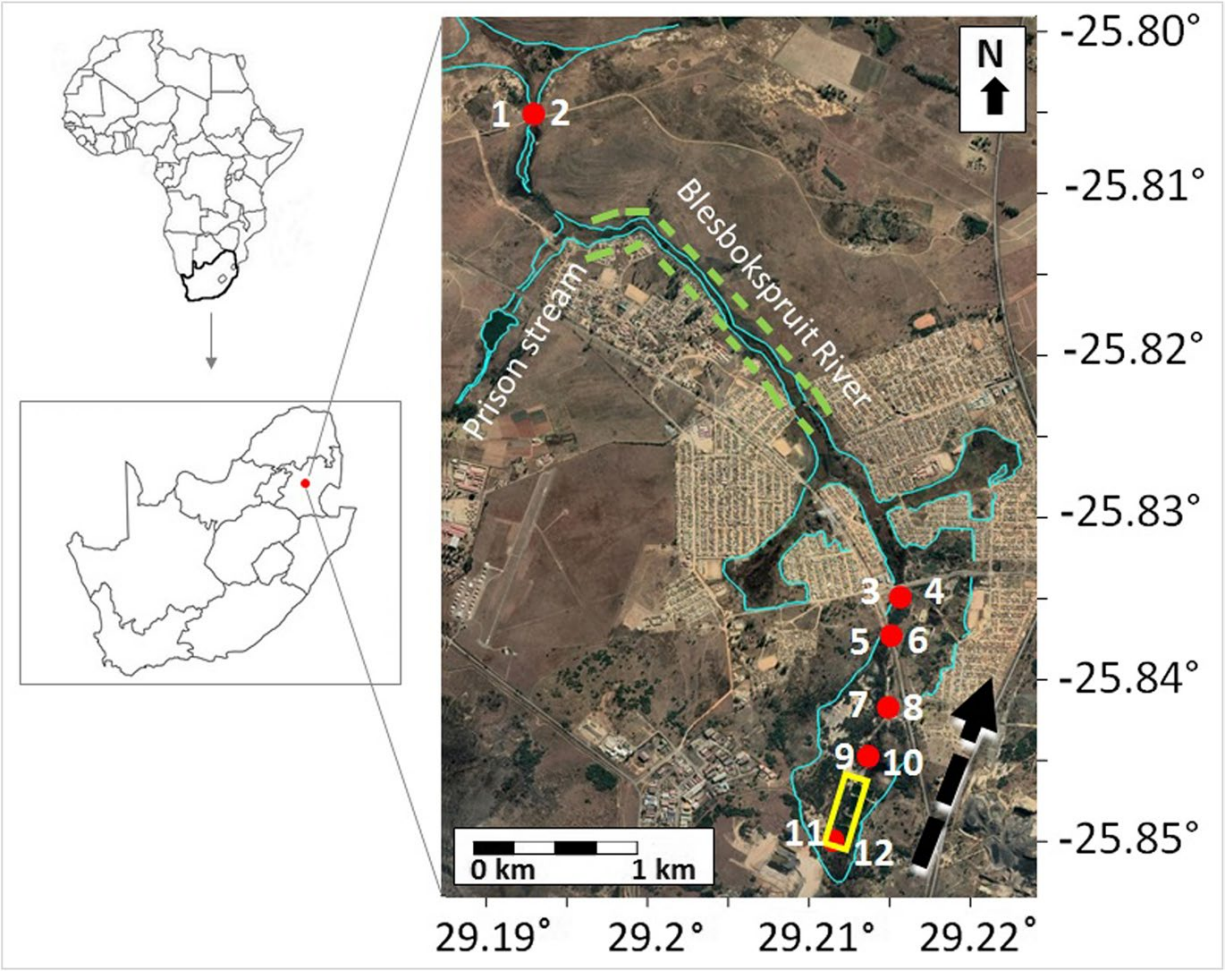
Localities (red dots) for overbank sediment sampling and ground hyperspectral data collection along the Blesbokspruit River, Mpumalanga, South Africa. Overbank sediment samples were collected at two sites roughly 5 m apart at each of the six different localities. Flow direction is indicated by the black, dashed arrow. Also shown is a wetland (green dash lines) and acid ponds (yellow rectangle)

The Blesbokspruit River is a low order stream that forms part of the Olifants River catchment in South Africa. It is located roughly 5 km NW of the town of Emalahleni in Mpumalanga province. Emalahleni is well known for its long history of coal mining (Bell et al., 2001). The Blesbokspruit River was chosen as an area of interest because (i) prior AMD-related studies have been conducted at this site (Bell et al., 2001; Netshitungulwana et al., 2013) and (ii) the site is not completely overgrown with vegetation and thus, it comprises several areas with exposed overbank sediments that are suitable for the measurement of ground spectra. The study site has four constructed acid pools located at the headwaters of the stream and a wetland located roughly 3 kms downstream of the acid pools (Fig. 1).

### Sample collection

Twelve overbank sediment samples (i.e., two samples roughly 5 m apart at six different localities) were collected along an approximately 6 km section of the Blesbokspruit River (Fig. 1). The collection of two overbank sediment samples roughly 5 m apart at each of the six different localities was in view of the knowledge that overbank sediment deposition rates show considerable spatial variability (Walling & He, 1998). The roughly 5 m separation from sample pairs at of the six different location was considered adequate considering that knowledge gained from this study will be imported to the subsequent analysis of 1-m spatial resolution airborne reflectance hyperspectral data that are available over the study area.

Samples were collected during autumn, one of the driest seasons of the year in the study area, during which moisture levels in overbank sediments are low and thus, ideal for the measurement of spectra in the field (Wu et al., 2005). The sampled section of the river was limited to the first 6 km downstream from the acid pools because of the neutralizing and diluting effect of the Prison tributary roughly 5 km downstream of the acid pools (Bell et al., 2001). Sample collection along the stream was also restricted by the presence of a wetland (Fig. 1) and sewage contamination from a neighboring informal settlement.

In geology and environmental sciences, more often than not, sample design and density are beyond the control of the researcher (Davis, 2002). Thus, geological and environmental data are occasionally sparse (e.g., *n* = 4 in Dragović et al., 2010; *n* = 6 in Mutiyar & Mittal, 2014; *n* = 3 in Baran & Tarnawski, 2015). In these situations, data must be collected where available and appropriate statistical analyses must be applied (Davis, 2002). In our case, the sparseness of data (due to financial, time, and field constraints) was addressed by applying statistical analyses that are appropriate for a small set of data and the uncertainty determined using confidence intervals (CIs) and statistical significance (*p*). While larger datasets are preferred over smaller ones (de Winter, 2013), they are not without their challenges. They can introduce arbitrary “spurious” correlations far exceeding the meaningful ones (Poppelars, 2015). As a result, the number of significant correlations are often exaggerated for larger datasets (Calude & Longo, 2017), thereby potentially impeding subsequent predictions. Here, we considered our samples *n* = 12 to be adequate for simple univariate regression (see the “Data analysis” section below) because, according to Van Voorhis and Morgan (2007), the absolute minimum sample size is *n* = 10 per predictor.

### Geochemical analysis

The sediment samples were air-dried at the base camp to limit possible changes in redox, and were passed through a 63 μm nylon sieve to separate the clay-and silt sized fraction. This size fraction has been considered the most important host of trace metals in soils and sediments (Förstner & Salomons, 1980). Nylon sieves and high density polyethylene storage bags were used to avoid possible contamination with metals targeted in this study (Zief & Mitchell, 1976). In the laboratory, the sieved samples were pretreated with reverse aqua regia (which excludes trace metals bound in the crystal lattice of primary minerals and releases the environmentally extractable proportions of trace metals) (Shahbazi & Beheshti, 2019) and then decomposed using microwave digestion for analysis by ICP-AES/MS. The samples were analyzed for major elements (Al, Fe, Si, and Mn) and trace metal (loid)s (Cu, Pb, Cd, and As) because they are often closely related to AMD (España, 2007; Nieto et al., 2007; Sengupta, 1993). The following were used in this study for quality assurance and quality control purposes: (i) field and analytical duplicates, (ii) procedural blanks, and (iii) soil certified reference materials (CRM). Analytical results for elements with precision of 20% or better (Ramsey, 1998) were retained for further data analysis.

### Mineralogical analysis

For XRD Rietveld analysis, overbank sediment samples were crushed and split, and subsamples were milled to obtain a size fraction less than 75 μm. Milling was necessary to ensure that (Buhrke et al., 1998): (i) crystallite orientations were randomized, (ii) there were adequate quantities of crystallites to yield a representative intensity distribution for a given sample, and (iii) adequate diffraction intensity was yielded to meet counting statistics. The milled samples were then analyzed using the Bruker D8 Advance diffractometer. The samples were not separated into various soil fractions prior to the XRD analysis to ensure that the results of this study will be consistent with the subsequent analysis of airborne hyperspectral data collected over the study area that is not discussed here but in a future publication.

### Ground spectral analysis

Using a portable ASD FieldSpec® 3 spectroradiometer, four ground spectral measurements were collected (because of the relative stability of measurements) at each of the twelve sample sites in six different localities (Fig. 1). The instrument used measures reflectance spectra across the VNIR–SWIR (350–2500 nm) region of the EM spectrum. Spectral data were measured in-situ (i.e., not dried prior to measurement) to ensure consistency with airborne hyperspectral data that are available over the study area. Spectra were measured under clear skies, with data collection restricted to 11 AM and 2 PM each day, when the sun was at or closest to its peak (Goetz, 2012). A field of view of 25° was used because this is considered best for ground spectral measurements (Janse et al., 2018) and a white reference panel (Spectralon) was used in between measurements as a baseline for the spectral measurements. The Spectralon was made of polytetrafluoroethylene and cintered halon (ASD Inc., 2009). This material is known to be nearly 100% reflective within the VNIR–SWIR wavelength range, scattering light uniformly in all directions within that wavelength range (ASD Inc., 2009).The ground spectra were captured using the RS^3^ software package included with the ASD FieldSpec® 3 spectroradiometer.

#### Preprocessing

**Fig. 2:**
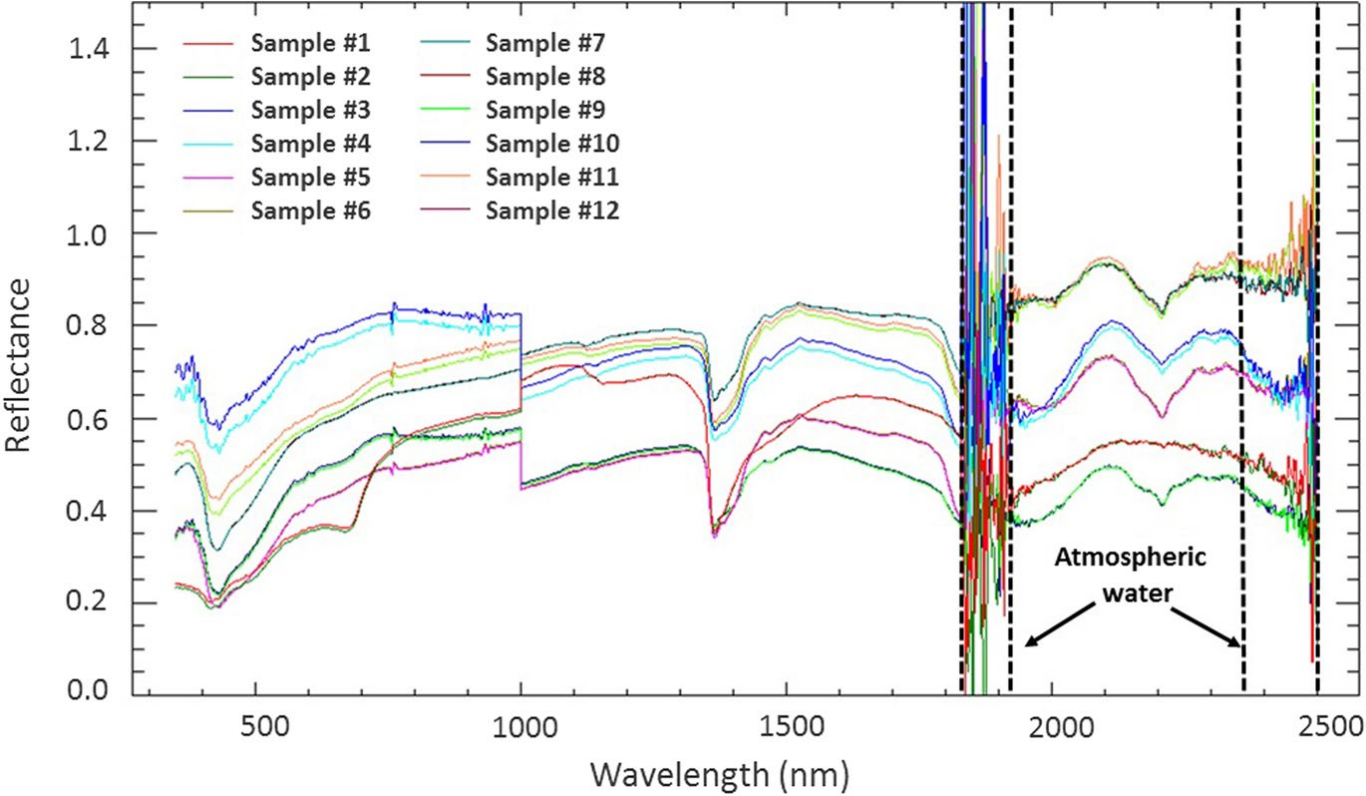
Median raw ground-derived spectra of overbank sediments at each of the 12 sample sites (Fig. 1) along the Blesbokspruit River, highlighting the wavelengths associated with atmospheric water

Because of the small number of measurements (*n* = 4) collected at each sample location, a combined spectral plot (Fig. 2) was generated using the median as an estimator of central tendency. The advantage of using the median over the mean is that (i) it is robust against outliers and (ii) it does not make any distributional assumptions, making it better suited for skewed data as is common in exploration and environmental geochemistry (Reimann & Filzmoser, 2000). There is a noticeable offset in spectra around 1000 nm (Fig. 2). This offset is a common problem associated with hyperspectral data when the same wavelength is measured by more than one sensor (Grillini et al., 2021). In our case, the offset is the result of the spectral overlap between the VNIR detector (measuring 350–1000 nm) and SWIR1 detector (measuring 1000–1800 nm) in the ASD FieldSpec® 3 spectroradiometer (ASD Inc., 2009).

**Fig. 3:**
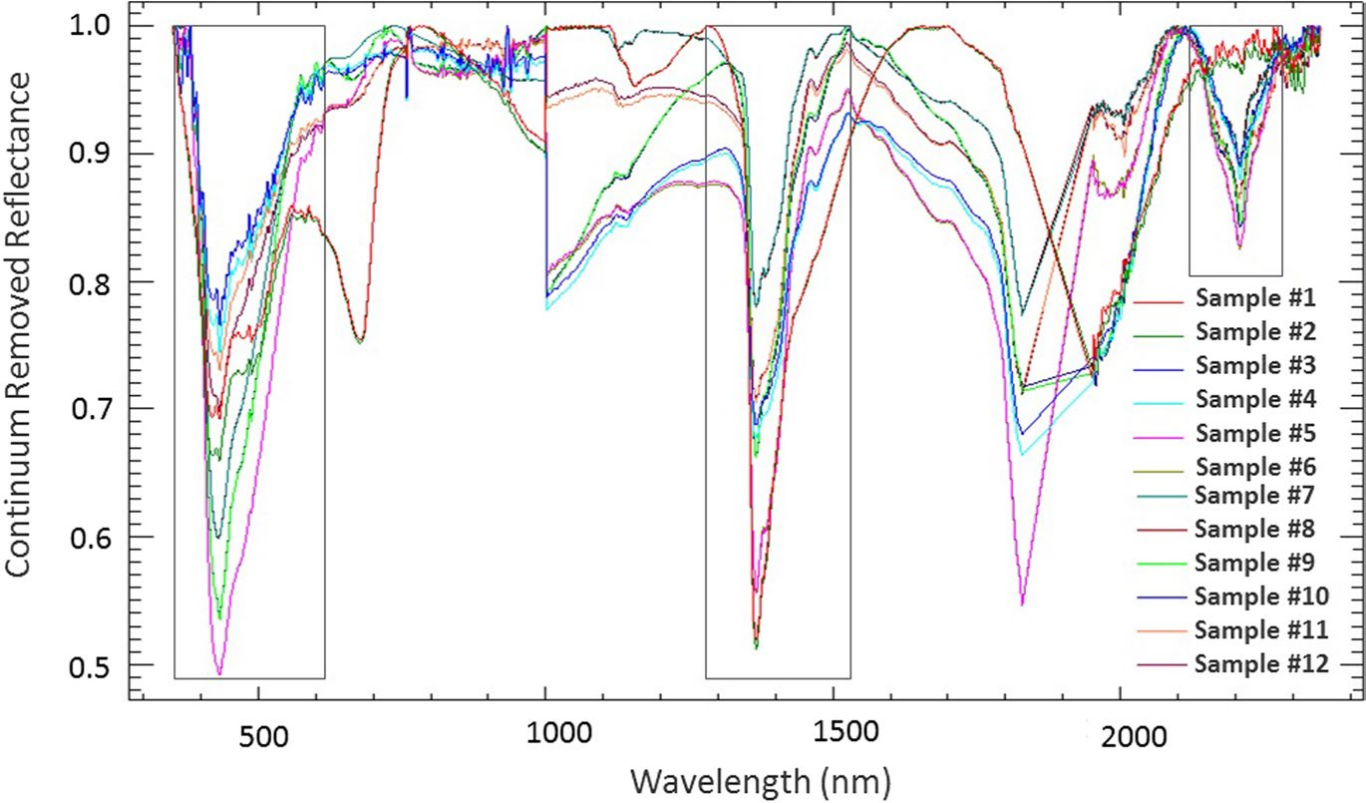
Continuum-removed ground spectra of overbank sediments at each of the 12 sample sites (Fig. 1) along the Blesbokspruit River, with wavelengths related to atmospheric water removed and the spectral subsets used to derive the SAFPs enclosed in black rectangles

Spectra between 1830 and 1930 nm, and between 2350 and 2500 nm (Fig. 2) were removed to exclude the noise associated with atmospheric water (Clevers et al., 2008; Pandit et al., 2010; Prasad et al., 2015). The remaining reflectance data were then enhanced using continuum-removal (CR), which is a widely used transformation in spectroscopy (Piroozfar et al., 2018; Prasad & Gnanappazham, 2016; Zhao et al., 2020). In CR analysis, the overall albedo of a reflectance curve (called the continuum) is removed, thereby scaling reflectance spectra to 100% when approaching the continuum (Van der Meer, 2004). Spectral absorption features do not occur at every wavelength within the VNIR–SWIR range but rather at certain wavelengths typically associated with oxides, clays, carbonates, sulfides, and organic matter (Malley & Williams, 1997). Here, changes in SAFPs, including absorption-band position, absorption-band depth (D), absorption-band width (W), and absorption-band asymmetry (S), were derived from the strongest SAFs in the CR spectra (Fig. 3) around 433 nm, which are known to be associated with lattice OH in goethite (Balsam & Wolhart, 1993), and around 1400 and 2200 nm, which are known to be associated with OH and Al–OH groups, respectively, related to kaolinite (Hunt & Ashley, 1979; Khunsa et al., 2017; Van der Meer, 1999).

#### Definition of the SAFPs

**Fig. 4:**
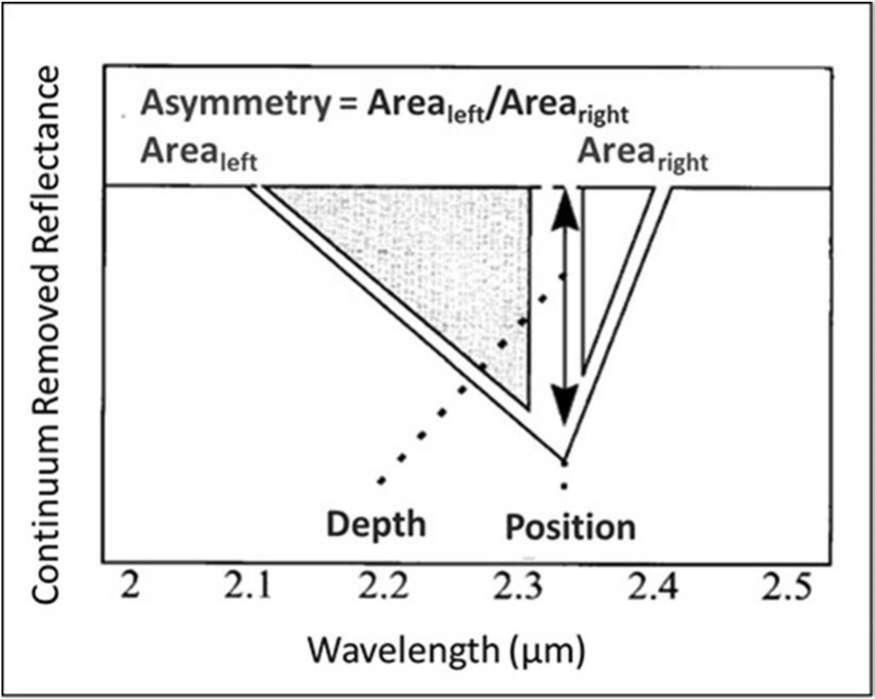
Definition of absorption-band position, depth, and asymmetry (modified after Van der Meer, 1999)

Absorption-band position, D, S, and W can be calculated from CR spectra according to Fig. 4. Absorption-band position is the wavelength corresponding with the minimum reflectance percentage over the wavelength range of the absorption feature (Van der Meer, 2004).

The D is typically defined relative to the hull/continuum (R_c_), as:

1 $$\textrm{D}=1\hbox{--} {\textrm{R}}_{\textrm{b}}/{\textrm{R}}_{\textrm{c}}$$

where R_b_ is the reflectance at the wavelength minimum and R_c_ is the reflectance of the continuum at the wavelength position of R_b_ (Van der Meer, 2004). The S is typically defined as:

2 $$\textrm{S}={\textrm{Area}}_{\textrm{left}}/{\textrm{Area}}_{\textrm{right}}$$

where the Area_left_ is the area from the starting point of the absorption feature to the minimum reflectance point and Area_right_ is the area from the minimum reflectance point to the shoulder (end point) of the absorption feature (Van der Meer, 2004). The W is typically defined as the area to the left and right of the minimum reflectance value/percentage of the absorption feature relative to the absorption depth (Van der Meer, 1999), thus:

3 $$\textrm{W}=\left({\textrm{Area}}_{\textrm{left}}+{\textrm{Area}}_{\textrm{right}}\right)/2\textrm{D}$$

#### Data analysis

Of the spectral data measured, all but the spectra in the subset around 2200 nm for samples no. 1 and 2 were used in further statistical analysis. Spectra measured around 2200 nm for samples no. 1 and 2 were excluded from the statistical analysis because they were very noisy and likely a result of the high percentage of local humidity (i.e., 24%) at the time and day of measurement. Because of the small number of overbank sediment samples (*n* = 12), correlations among the CR SAFPs were determined using Spearman’s rank correlation analysis, which is suitable for smaller datasets and is robust against outliers (Reimann & Filzmoser, 2000). The reliability of the correlations is expressed by the statistical significance (*p*) and confidence intervals (CIs). Although the trace metal concentrations are compositional data (Buccianti & Pawlowsky-Glahn, 2005; Pawlowsky-Glahn & Egozcue, 2006), they were not subjected to log-ratio transformation because the data per element were correlated with spectral data which do not come from the same, closed composition, thus, rendering them independent (Reimann & de Caritat, 2017). However, the trace metal concentration data were *ln*-transformed to improve the normality of the data prior to further analysis (Reimann & Filzmoser, 2000). The relationships between trace metal concentration data (as target variables) and spectral data (as predictor variables) can be modeled using linear regression analysis (i.e., one predictor variable (or regressor) and one target variable (or regressand). However, multiple linear regression analysis is unsuitable when predictor variables show high collinearity, as is typical of spectral data (Van der Meer & Jia, 2012). In addition, the resulting models are often too complex to understand (Wu et al., 2005).

Here, SAFPs which showed the strongest correlations with trace metal contents were used to establish univariate regression models, thereby, satisfying the “one in ten” rule of thumb for the number of samples required per predictor variable in regression analysis (Austin & Steyerberg, 2015; Harrell Jr., 2001; Steyerberg, 2009). Calibration regression models were evaluated using the coefficient of determination (R^2^) and the standard error of estimation (SEE) as fitness indicators. The goodness of fit increases as R^2^ values approach 1 while the uncertainty of the calibration models decrease as SEE values approach 0. “Predicted” concentrations were calculated using the regression equation obtained for each of the calibration regression models. Predicted concentrations in *ln* were then back-transformed to normal values for comparison with measured trace metal concentrations. The predictive capacity of each of the regression models was evaluated using the leave-one-out cross-validation (LOO-CV) method, which is suitable for very small datasets (Yadav & Shukla, 2016). Here, a single data point was removed from the dataset and the regression analysis performed using the remaining data points. The resulting model was then used to predict the removed data point and the squared error (SE) was calculated for the predictive models. This process was repeated until each of the data points has been removed and used for cross-validation. The prediction capacity was determined by calculating the root mean squared error of prediction of cross-validation (RMSEP_CV_). The stability of the predictive models was evaluated using the Chow test (Chow, 1960), which examines the equality of regression coefficients (i.e., slope and intercept) across subsets of the data and returns a significant result if the coefficients are statistically different (Sotirakopoulos et al., 2015).

## Results and discussion

### Mineralogy and geochemistry

Table 1 shows the mineralogical and trace metal composition of the overbank sediment samples collected from the study site. The results of XRD Rietveld analysis showed that the overbank sediments in the study site contained mainly quartz (95–100%) and kaolinite (0–5%). Thus, they can be considered as sediments with sandy texture. The major element composition of the overbank sediment samples can be summarized according to decreasing median concentrations (ppm) as: Al (44719.56) > Fe (25670.24) > Si (3459.86) > Mn (212.17). The trace metal composition of the overbank sediment samples can be summarized according to decreasing median concentrations (ppm) as: Cu (31.77) > Pb (25.21) > As (7.40) > Cd (0.03). The major element composition of the overbank sediments suggests that the < 63 μm fraction of the overbank sediments is largely dominated by Al and Fe oxides and oxyhydroxides, with lower proportions of clay (indicated by Si) and minor proportions of Mn oxides and oxyhydroxides.

**Table 1:** Mineralogical (%) and trace metal composition (ppm) of Blesbokspruit River overbank sediment samples, as determined by XRD Rietveld analysis and ICP-AES/MS, respectively

Sample #	% Quartz	% Kaolinite	Si	Al	Fe	Mn	Pb	Cd	As	Cu
1	99	1	4018.99	34055.15	60397.83	810.81	19.30	0.03	8.56	39.58
2	99	1	2995.00	42604.86	63870.49	958.88	23.63	0.03	8.94	50.81
3	100	0	3481.57	35141.18	19537.25	199.16	22.90	0.01	5.24	27.07
4	99	1	3718.11	41685.31	26825.22	325.12	26.78	0.05	6.52	31.59
5	95	5	3814.73	51827.09	26902.85	262.41	31.67	0.26	8.29	42.81
6	96	4	2968.00	46834.26	25172.25	189.48	35.76	0.30	8.61	46.68
7	99	1	3472.74	51362.93	26168.22	112.34	23.45	0.02	5.07	27.42
8	98	2	3709.28	57907.06	33456.19	238.30	27.33	0.07	6.36	31.94
9	99	1	3210.44	32634.63	17837.61	99.22	51.22	0.02	11.15	31.05
10	100	0	3446.98	39178.77	25125.58	225.18	47.34	0.06	12.44	35.57
11	100	0	2005.81	83014.95	12225.91	126.44	22.86	0.01	2.64	23.93
12	100	0	2598.00	72826.09	11932.37	103.51	20.36	0.02	2.70	23.85
Median	99	1	3459.86	44719.56	25670.24	212.17	25.21	0.03	7.40	31.77

Correlations among the derived goethite- and kaolinite-related SAFPs and *ln*-transformed trace metal contents are shown in Table 2. The rationale for trace metal predictions is based on the knowledge that iron oxide minerals (Parker et al., 2007; Webster et al., 1998), such as goethite, and clay minerals (Uddin, 2017; Ugwu & Igbokwe, 2019), such as kaolinite, play significant roles in trace metal attenuation in aquatic environments, largely via adsorption processes. According to Table 2, Cd showed strong (*r* > 0.7) and significant (CI > 95%) correlation with only the goethite-related SAFP (Depth_433_). This strong correlation is supported by Covelo et al. (2007) who determined that Cd was preferentially adsorbed (and retained) by Fe-oxides, compared to clays, in soils. Pb showed strong (*r* > 0.7) and significant (CI > 95%) correlations with both the goethite-related SAFPs (Depth_433_ and Width_433_) and the kaolinite-related SAFP (Width_2208_). These strong correlations are consistent with Moreno et al. (2006) who found that iron oxide and clay (the most significant of which was kaolinite) contents played important roles in the adsorption of Pb. Arsenic appeared strongly correlated (*r* > 0.7) with both goethite-related (Asym_433_) and kaolinite-related (Asym_2208_) SAFPs. While the strong and significant correlation with goethite is expected (Kumpiene et al., 2008; Palansooriya et al., 2020), the strong correlation with kaolinite (Asym_2208_) is consistent with Choe et al. (2009) and Piroozfar et al. (2018) and may be attributed to strong complexes between As and (i) octahedrally coordinated aluminum- (Halter & Pfeifer, 2001) and (ii) Fe and Al hydroxide coatings on kaolinite surfaces (Goldberg, 2002). Similar to As, Cu is strongly correlated (*r* > 0.7) with both goethite- (Asym_433_) and kaolinite-related (Depth_1366_, and Width_1366_) SAFPs. Copper is commonly associated with Fe oxides (Kabata-Pendias & Pendias, 2000; Kumpiene et al., 2008) and similar to As, has shown a strong affinity for kaolinite when it is coated with Fe hydroxides (González-Costa et al., 2017; Osei & Singh, 2000; Zhuang & Yu, 2002), as is applicable in the study area.

**Table 2:** Correlations between the derived SAFPs and *ln*-transformed trace metal concentrations. Strong (*r* > = 0.7) and significant (*p* < 0.05) correlations are shown in bold

*n* = 12	*ln*.Pb	*ln*.Cd	*ln*.As	*ln*.Cu
Depth_433_	0.7**	0.7**	0.6*	0.5
Asym_433_	−0.2	−0.4	−0.7*	−0.8**
Width_433_	−0.7*	−0.2	−0.5	−0.1
Depth_1366_	0.1	0.4	0.5	0.8**
Asym_1366_	−0.6*	−0.3	−0.5	−0.3
Width_1366_	−0.3	−0.1	−0.7	−0.7**
Depth_2208_	0.6*	0.4	−0.0	−0.2
Asym_2208_	−0.4	−0.4	−0.7**	−0.6
Width_2208_	−0.8**	−0.2	0.0	−0.3

*Correlation is significant at the 0.05 level (2-tailed)

**Correlation is significant at the 0.01 level (2-tailed)

### Regression modeling

#### Model calibration

Inputs to the univariate regression modeling were based on correlations of the SAFPs with the *ln*-transformed trace metal concentrations (Table 2). Table 3 shows the R^2^ and SEE of the calibration models obtained for Pb, Cd, As, and Cu. The SEE generally provides a better estimate of a model’s predictive accuracy, compared to the R^2^, because it is a measure of the actual distance of the data points from the regression line on average (Frost, 2023a). Thus, according to the SEE, the best performing model was obtained for Cu (SEE = 0.15), followed by Pb (SEE = 0.21), As (SEE = 0.31), and Cd (SEE = 0.81).

**Table 3:** Goodness of fit (R^2^) and uncertainty (SEE) of the univariate calibration regression models for the SAFPs and trace metal contents

Metal	Predictor	R^2^	SEE*
Pb	Width_2208_	0.61	0.21
Cd	Depth_433_	0.56	0.81
As	Asym_2208_	0.70	0.31
Cu	Depth_1366_	0.69	0.15

*Standard error of estimation

#### Model evaluation

Table 4 shows the regression equations, SE, RMSEP_CV_ and Chow statistic (*p*) for Pb, Cd, As, and Cu, using the LOO-CV method. According to the Chow statistic (Table 4), regression coefficients generated by CV, for each of the trace metals, were stable (*p* > 0.05) and thus, robust, in spite of the small sample size. According to Table 4, the lowest RMSEP was obtained for Cd (0.06), followed by As (1.70), Cu (2.50), and Pb (4.30). However, when the RMSEP is compared with the concentration range of each of the metals, the RMSEP obtained for Cd represents ~ 25% of the population; for As, it represents ~ 17% of the population; for Cu, it represents ~ 9% of the population and for Pb, it represents ~ 14% of the population. Thus, Cu was the most accurately predicted while Cd was the least accurately predicted.

**Table 4:** Regression equations, SE and RMSEP_CV_ and Chow statistic for Pb, Cd, As, and Cu, using the LOO-CV method

Metal	Sample #s included in CV^a^	Regression equation^b^	SE^c^ (y - ŷ)^2^	RMSEP_CV_^d^	Chow test (*p*)
Pb	2, 3, 4, 5, 6, 7, 8, 9, 10	*y* = 0.60*x* + 12.2	19.7	4.30	0.20
	1, 3, 4, 5, 6, 7, 8, 9, 10	*y* = 0.60*x* + 13.0	3.8		
	1, 2, 4, 5, 6, 7, 8, 9, 10	*y* = 0.61*x* + 11.6	0.5		
	1, 2, 3, 5, 6, 7, 8, 9, 10	*y* = 0.62*x* + 11.6	3.9		
	1, 2, 3, 4, 6, 7, 8, 9, 10	*y* = 0.59*x* + 12.4	8.4		
	1, 2, 3, 4, 5, 7, 8, 9, 10	*y* = 0.59*x* + 12.6	2.5		
	1, 2, 3, 4, 5, 6, 8, 9, 10	*y* = 0.60*x* + 12.0	73.4		
	1, 2, 3, 4, 5, 6, 7, 9, 10	*y* = 0.54*x* + 13.2	67.7		
	1, 2, 3, 4, 5, 6, 7, 8, 10	*y* = 0.65*x* + 9.8	3.4		
	1, 2, 3, 4, 5, 6, 7, 8, 9	*y* = 0.69*x* + 8.5	4.6		
Cd	2, 3, 4, 5, 6, 7, 8, 9, 10, 11, 12	*y* = 0.35*x* + 0.03	6.59E-05	0.06	0.18
	1, 3, 4, 5, 6, 7, 8, 9, 10, 11, 12	*y* = 0.36*x* + 0.03	4.55E-05		
	1, 2, 4, 5, 6, 7, 8, 9, 10, 11, 12	*y* = 0.35*x* + 0.03	5.10E-04		
	1, 2, 3, 5, 6, 7, 8, 9, 10, 11, 12	*y* = 0.35*x* + 0.03	3.98E-05		
	1, 2, 3, 4, 6, 7, 8, 9, 10, 11, 12	*y* = 0.35*x* + 0.03	0.02		
	1, 2, 3, 4, 5, 7, 8, 9, 10, 11, 12	*y* = 0.39*x* + 0.02	0.03		
	1, 2, 3, 4, 5, 6, 8, 9, 10, 11, 12	*y* = 0.37*x* + 0.02	1.45E-04		
	1, 2, 3, 4, 5, 6, 7, 9, 10, 11, 12	*y* = 0.36*x* + 0.03	2.84E-04		
	1, 2, 3, 4, 5, 6, 7, 8, 10, 11, 12	*y* = 0.39*x* + 0.02	1.82E-04		
	1, 2, 3, 4, 5, 6, 7, 8, 9, 11, 12	*y* = 0.36*x* + 0.02	2.38E-04		
	1, 2, 3, 4, 5, 6, 7, 8, 9, 10, 12	*y* = 0.35*x* + 0.03	3.16E-04		
	1, 2, 3, 4, 5, 6, 7, 8, 9, 10, 11	*y* = 0.35*x* + 0.03	2.13E-04		
As	2, 3, 4, 5, 6, 7, 8, 9, 10	*y* = 0.47*x* + 3.3	0.2	1.70	0.06
	1, 3, 4, 5, 6, 7, 8, 9, 10	*y* = 0.45*x* + 3.5	0.0		
	1, 2, 4, 5, 6, 7, 8, 9, 10	*y* = 0.42*x* + 3.5	1.5		
	1, 2, 3, 5, 6, 7, 8, 9, 10	*y* = 0.42*x* + 3.7	2.0		
	1, 2, 3, 4, 6, 7, 8, 9, 10	*y* = 0.49*x* + 3.0	0.2		
	1, 2, 3, 4, 5, 7, 8, 9, 10	*y* = 0.45*x* + 3.3	0.0		
	1, 2, 3, 4, 5, 6, 8, 9, 10	*y* = 0.52*x* + 3.2	4.6		
	1, 2, 3, 4, 5, 6, 7, 9, 10	*y* = 0.62*x* + 2.6	4.2		
	1, 2, 3, 4, 5, 6, 7, 8, 10	*y* = 0.32*x* + 4.7	8.6		
	1, 2, 3, 4, 5, 6, 7, 8, 9	*y* = 0.32*x* + 4.7	8.1		
Cu	2, 3, 4, 5, 6, 7, 8, 9, 10, 11, 12	*y* = 0.66*x* + 10.6	8.2	2.50	0.48
	1, 3, 4, 5, 6, 7, 8, 9, 10, 11, 12	*y* = 0.68*x* + 10.6	32.9		
	1, 2, 4, 5, 6, 7, 8, 9, 10, 11, 12	*y* = 0.71*x* + 9.7	3.4		
	1, 2, 3, 5, 6, 7, 8, 9, 10, 11, 12	*y* = 0.70*x* + 9.7	0.6		
	1, 2, 3, 4, 6, 7, 8, 9, 10, 11, 12	*y* = 0.72*x* + 9.4	7.1		
	1, 2, 3, 4, 5, 7, 8, 9, 10, 11, 12	*y* = 0.77*x* + 7.9	7.8		
	1, 2, 3, 4, 5, 6, 8, 9, 10, 11, 12	*y* = 0.70*x* + 10.8	4.7		
	1, 2, 3, 4, 5, 6, 7, 9, 10, 11, 12	*y* = 0.70*x* + 10.7	1.0		
	1, 2, 3, 4, 5, 6, 7, 8, 10, 11, 12	*y* = 0.72*x* + 9.0	0.2		
	1, 2, 3, 4, 5, 6, 7, 8, 9, 11, 12	*y* = 0.72*x* + 9.5	0.3		
	1, 2, 3, 4, 5, 6, 7, 8, 9, 10, 12	*y* = 0.76*x* + 7.5	3.4		
	1, 2, 3, 4, 5, 6, 7, 8, 9, 10, 11	*y* = 0.76*x* + 7.5	3.5		

^a^Cross-validation

^b^*y* refers to metal, *x* refers to SAFP predictor

^c^Squared error

^d^Root mean squared error of prediction of cross-validation

**Fig. 5:**
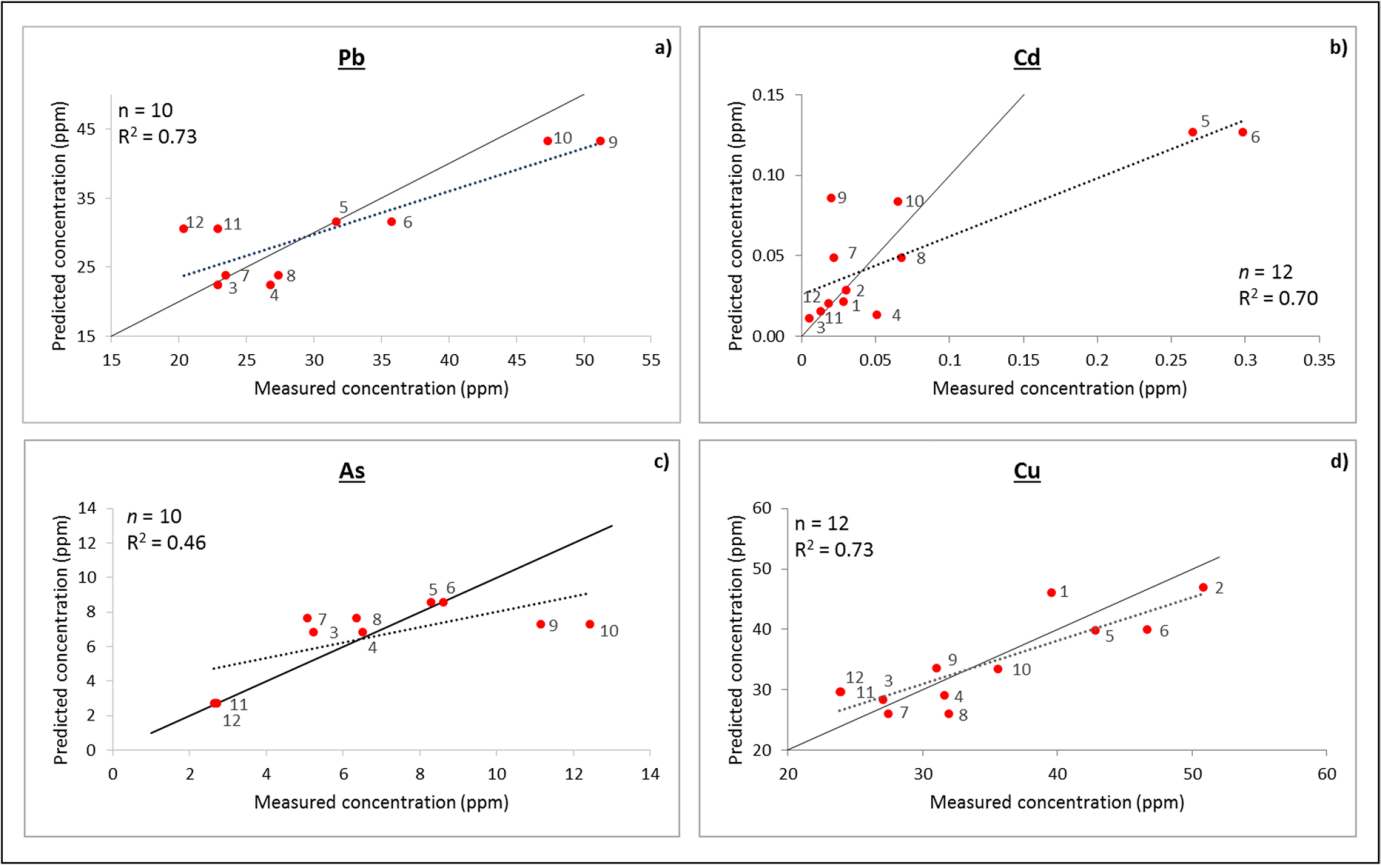
Measured vs. predicted concentrations of **a** Pb, **b** Cd, **c** As, and **d** Cu in overbank sediments in the study area (red dots with sample identification). The 1:1 control lines are shown in solid black and the regression lines are shown in dotted grey line

Scatterplots depicting measured vs. predicted trace metal (Pb, Cd, As, and Cu) contents were generated as part of the predictive model evaluation process (Fig. 5). Data points appeared mostly scattered in pairs (generally corresponding with the 5-m-spaced sample pairs). According to Fig. 5, the highest R^2^ was obtained for Cu (R^2^ = 0.73) and Pb (R^2^ = 0.73), followed by Cd (R^2^ = 0.70) and As (R^2^ = 0.46). The R^2^ values obtained for Pb (Fig. 5a) and Cu (Fig. 5d) are in good agreement with the distribution of points around their 1:1 control lines. However, the regression line obtained for Cd (Fig. 5b) showed significant deviation from the 1:1 control line, suggesting that its high R^2^ is likely strongly influenced by the outliers at ~ 0.25 ppm. When the outliers are removed from the regression analysis, the R^2^ lowers substantially from 0.7 to 0.17. This suggests that the Cd model is inappropriate for predictions and thus, is a limitation of this study.

The RMSEP (Table 4), which generally shows a high sensitivity to outliers (Frost, 2023b), is a seemingly unsuitable estimation of model performance, especially for Cd. In addition, it was strongly controlled by the concentration range of the training dataset (i.e., Cd had the lowest RMSEP because its training dataset had the lowest mean) and does not agree with the regression line obtained for Cd (Fig. 5b). In contrast, the SEE (Table 3), showed better consistency with the regression line obtained for Cd, compared to the RMSEP, and thus, is a more suitable estimate of model accuracy.

## Conclusions

Conventional geochemical methods of investigating trace metal contents in soils and sediments can be very costly and time-consuming, especially when conducted on large scales. This study served as a first attempt to predict potentially toxic trace metals in overbank sediments of the Witbank Coalfield using VNIR–SWIR spectroscopy and the distinct SAFPs of mineral-bound trace metals. We determined the following:

1. The strongest calibration models were obtained using Depth_433_, Depth_1366_, Width_2208_, and Asym_2208_ as trace metal predictors. The association of these predictors with goethite and kaolinite suggests that these minerals play an important role in trace metal attenuation and prediction in the study area.
2. Of the trace metals analyzed, Cu and Pb were the easiest to predict while As and Cd were harder to predict.
3. The RMSEP was highly sensitive to outliers, as well as the concentration ranges of the analyzed metals. Thus, it provided a seemingly inappropriate estimate of predictive model accuracy. In contrast, the SEE appeared more consistent with the regression lines obtained for the predictive models and thus, was a better estimate of the predictive accuracy, overall.

Although the data analysis was tailored to the small sample size, it remains a limitation of this work. This study, therefore, serves mainly as a proof of concept. Additional research using a larger dataset and more complex multivariate regression analysis is warranted to improve the validation of the predictive models. Following improved validation, ground reflectance spectroscopy could prove to be a valuable screening tool for detecting trace metal concentrations in overbank sediments with significant Fe oxides and clays, as a precursor to more in-depth sampling and geochemical analyses.

## Data Availability

The data that support the findings of this study are available from the corresponding author, Jamie-Leigh Robin Abrahams, upon reasonable request.
